# Improved Step-by-Step qPCR Method for Absolute Telomere Length Measurement

**DOI:** 10.3390/mps9010022

**Published:** 2026-02-05

**Authors:** Ekaterina Sergeevna Arshinova, Nataliia Sergeevna Karpova, Olga Leonidovna Terekhina, Malik Nurbekov, Maria Ivanovna Burtovskaya

**Affiliations:** Federal State Budgetary Institution “Research Institute of Pathology and Pathophysiology”, 125315 Moscow, Russia; nataliiakarpova.sp@gmail.com (N.S.K.); olga.terekhina1507@gmail.com (O.L.T.); m.burtovskaya@gmail.com (M.I.B.)

**Keywords:** biological aging, telomere length, quantitative PCR, telomere, protocol, method

## Abstract

Telomere length is a crucial marker of cellular aging and genomic stability, with significant implications for age-related diseases and cancers. This study introduces an improved quantitative PCR (qPCR) method for measuring absolute telomere length, addressing the need for accurate and high-throughput assessment in both clinical and research settings. Novel primers were designed for the single-copy gene interferon beta (*IFNB1*) to serve as an internal control, alongside a series of single-stranded oligonucleotide standards to establish a calibration curve. This approach allows for precise quantification of telomere length in kilobases per single copy gene copy number per chromosome. We validated this method using DNA samples from peripheral blood and buccal swabs from 17 healthy human volunteers, as well as umbilical cord blood from 9 healthy newborn babies, demonstrating its high linearity and reproducibility. Our findings indicate that this improved qPCR technique provides a rapid, cost-effective, and accurate means of measuring absolute telomere length, thereby facilitating large-scale studies and enhancing clinical diagnostics related to telomere biology.

## 1. Introduction

Telomeres, the short tandem repeats of DNA with DNA–protein structures found at both ends of each chromosome, play a vital role in securing the genome from degradation and interchromosomal fusion [1]. The shortening of telomere length is regarded as a significant and debated biomarker of aging, as it influences the essential biological processes associated with aging, such as cellular senescence and genomic instability [2]. Measuring telomere length is important in biomedical, epidemiological, and public health research to understand the cumulative effects of environmental exposures and life experiences and to assess the risk of major diseases [3]

Although for telomere length measurement a wide range of methods (Supplementary Material S1) have been developed, quantitative or real-time polymerase chain reaction (PCR) protocol is seen as a less time-consuming, more financially affordable and flexible method, which requires a small concentration of DNA samples (~20 ng) and can be performed for large-scale studies to obtain absolute and relative telomere length (rTL) [4]. However, this rTL method offers a relative measurement of the total telomere length in relation to a single-copy gene ratio and does not give insights into the distribution of telomere lengths on a specific single chromosome [5].

To ensure high precision and accuracy, careful methodological analysis is crucial due to inconsistencies in qPCR measurements arising from issues like incorrect single copy gene selection, flawed oligomer and primer design, and variations in raw data calculation and analysis [4]. High-quality DNA, telomere and single-copy gene primers, a master mix, and a well-calibrated qPCR instrument are essential for accurate qPCR-based rTL analysis [6].

rTL and absolute telomere length (aTL) measurements differ significantly in their approach and utility. The rTL, typically assessed via qPCR, provides a $\frac{T}{S}$ (where *T* is Telomere and *S* is Single Copy Gene) ratio relative to a known housekeeping DNA sample, offering an average telomere length per genome. The aTL quantifies telomere length in base pairs with synthetic oligomer DNA standard calibration curves, enabling more accurate tracking of changes and revealing critical insights into telomere dynamics in aging, cancer, and other diseases [7].

The peripheral blood as a DNA experimental sample is favored for its easy accessibility, high renewability, and standardized methods for DNA extraction. It is also a valuable source for telomere length longitude observation studies as telomere length in peripheral blood changes over time due to genetics, environmental factors, lifestyle choices, and variations among blood cell types. The dynamic nature of telomere length in peripheral blood, including both shortening and elongation, emphasizes the need to account for these variations when using telomere length as a health and aging biomarker [8].

**Figure 1:**
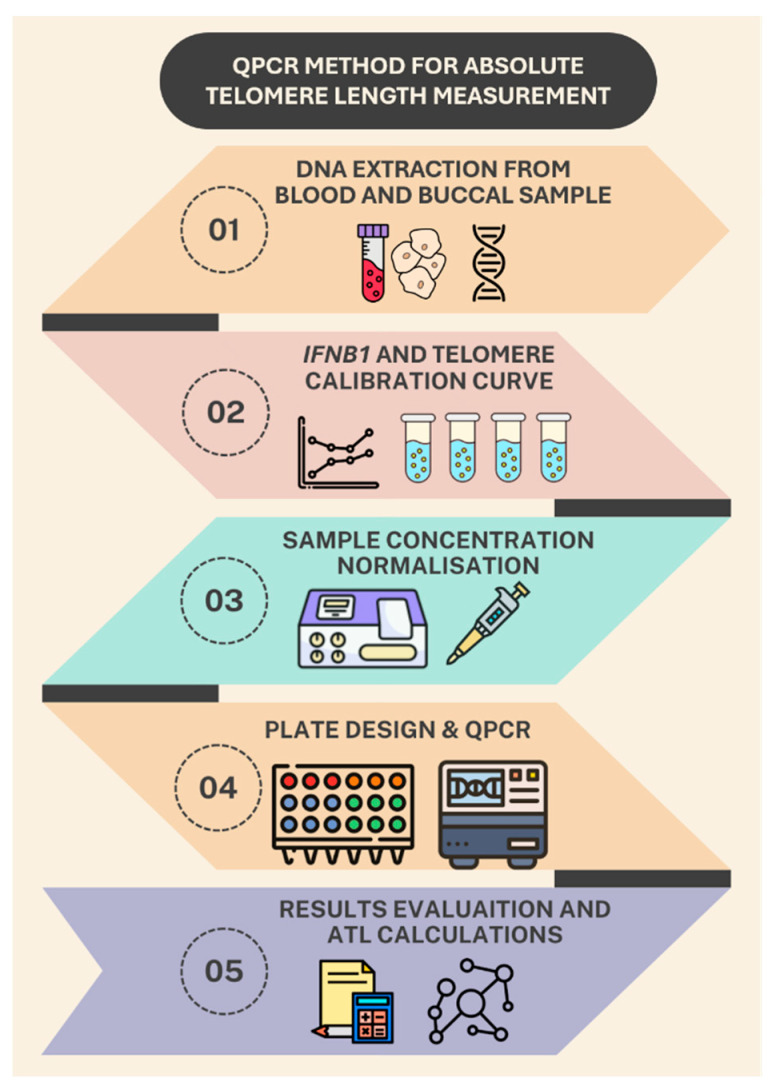
aTL qPCR Method Step by Step Overview.

In this study an improved qPCR method is introduced for measuring absolute telomere length. By designing new primers for the single-copy gene interferon beta (*IFNB1*) and utilizing single-stranded oligonucleotide standards, we establish a precise calibration curve that enables the quantification of telomere length in kilobases per single copy gene copy number per chromosome. Validation of the updated methodological approach will be carried out on an expanded panel of samples, including DNA from peripheral blood and their buccal epithelium of healthy adult volunteers, as well as umbilical cord blood of newborns. The use of two types of tissues—hematopoietic and epithelial—will demonstrate the versatility of the method for DNA analysis of various cellular origins and confirm the absence of tissue-specific artifacts. In turn, a comparative analysis of adult and newborn samples will show the applicability of the method for the correct assessment of age-related variability in telomere length. This improved technique promises to provide a rapid, cost-effective, and accurate means of assessing absolute telomere length, thereby facilitating large-scale studies and enhancing clinical diagnostics related to telomere biology. Overview of the aTL Method for Telomere qPCR is illustrated in Figure 1.

By advancing the precision and efficiency of telomere length measurement, this study seeks to contribute significantly to the understanding and diagnosis of age-related diseases and cancers, ultimately paving the way for improved clinical outcomes.

## 2. Experimental Design

### 2.1. DNA Extraction and Storage

All samples of peripheral blood, cord blood, and buccal swabs were collected from healthy volunteers without chronic diseases individually in commercial labs. DNA was extracted from whole blood cells, cord blood, and buccal swab samples and purified with the Extract DNA Blood and Cells (Evrogen, Moscow, Russia, BC111M), in accordance with the instructions of the manufacturer following safety rules working with hazardous materials. Buccal swabs collect epithelial cells from the inner cheek lining, which are relatively accessible but may yield variable DNA quality and quantity. Umbilical cord blood was collected from women immediately after they gave birth to their babies. Informed consent for the collection and use of cord blood samples was obtained from all mothers prior to delivery. For peripheral blood and buccal swabs, consent was obtained directly from the adult volunteers. The high-molecular DNA was stored at −20 °C. The quantity and quality of the isolated DNA and oligonucleotide dilutions were assessed with the NanoDrop 1000 spectrophotometer (Thermo Fisher Scientific, Wilmington, DE, USA.) in dsDNA 50 mode and ssDNA mode accordingly. When extracting DNA from human blood, umbilical cord blood, and buccal swab samples using the Extract DNA Blood and Cells (Evrogen, Moscow, Russia, BC111M), the protocol is specifically designed to isolate and purify genomic DNA, ensuring that the final product contains only DNA and not mRNA or cDNA. The kit utilizes denaturing agents and optimized buffers that lyse cells, denature proteins, and selectively bind DNA to a purification matrix, effectively removing RNA, including mRNA, and preventing the synthesis of cDNA, which would require reverse transcription enzymes not present in the DNA extraction workflow or further in PCR protocol.

### 2.2. Primers and Oligomers

All primers underwent HPLC purification, while oligomers were purified using PAGE (Evrogen, Moscow, Russia, SP001). Telomere and Exon *IFNB1* oligomers were diluted and utilized as single-stranded antisense standards, adapting to laboratories lacking double-chain nucleotide synthesis capabilities (Supplementary Material S2). In previous studies, the plasmids were utilized as uniform templates that help control for variations in PCR efficiency across different plates and experiments [9,10]. In this study, we ran the analysis without plasmid DNA (pBR322) using the comparative *IFNB1* aTL no plasmid protocol. Plasmids affect PCR outcomes by altering DNA structure leading to underestimation in qPCR quantification as it can hinder PCR amplification, impacting the accuracy of standard curves and overall quantification results [11]. The sequence for telomere primers (FTM, RTM) and oligomer standards was taken from a previous study [10]. The primers Fexon *IFNB1*, Rexon *IFNB1* (Table 1), and Standard Oligos of *IFNB1* were specifically designed to address dimer formation issues.

All stock primers with 100 μM were 10× diluted for the working solution. Their sequences target the exon coding region of the *IFNB1* gene (Table 2).

The oligomer stock, initially at 100 μM concentration, underwent serial 10-fold dilutions to form a calibration curve. For the qPCR assay, standards 4–8 were used for telomeres, while standards 5–9 were employed for *IFNB1* measurements (Table 3). Telomeres are amplified earlier compared to a single-copy gene during PCR, thus more concentrated standards were used for telomeres and less concentrated ones were utilized for *IFNB1*.

### 2.3. qPCR Conditions

**Figure 2:**
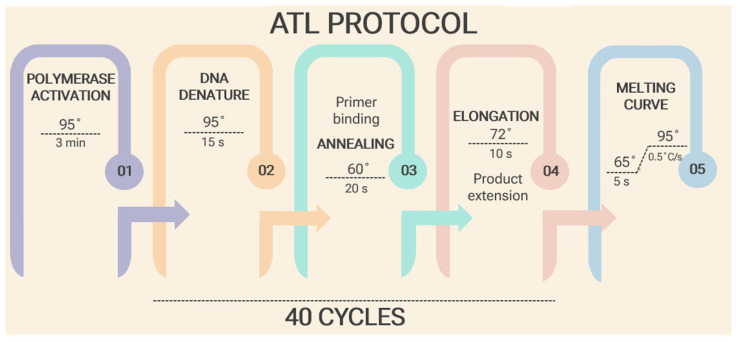
aTL Protocol for Telomere qPCR. The dashed line indicates the amplification steps (02–04) repeated for 40 cycles.

Amplification was carried out in the CFX96 Touch Real-Time PCR System (Bio-Rad, Laboratories, Hercules, CA, USA) with the subsequent thermocycling parameters for telomere and single copy gene: preincubation at 95 °C for 3 min; then 40 cycles including denaturation at 95 °C for 15 s, at 60 °C for 20 s, at 72 °C for 10 s (signal acquisition), and 60 °C for 30 s (melt curve) (Figure 2). Protocol step temperatures were set up according to primer design conditions. The obtained data was examined using the Bio-Rad CFX Manager software, version 3.0 (Bio-Rad Laboratories, Hercules, CA, USA).

### 2.4. qPCR MasterMix and Plate Preparation

**Figure 3:**
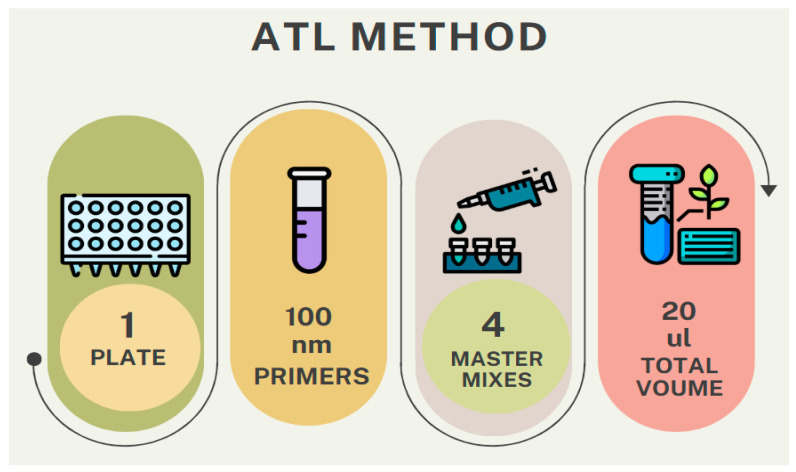
Overview of the aTL Method for Telomere PCR.

This qPCR protocol is designed for a total reaction volume of 20 μL per well. The master mix composition includes 0.2 μL of each primer at 100 μM concentration, 4 μL of Evrogen 5X qPCRmix-HS SYBR (Evrogen, Moscow, Russia, PK147L), and 14.6 μL of nuclease-free water. For standard reactions, 1 μL of template is used, while sample reactions use 4 μL of template with a concentration ranging from 18 ng/μL to 22 ng/μL, as the method is sensitive to variations in template quantity. The purity and concentration of the extracted DNA were assessed, with the molecular weight considered to be approximate, as precise determination was not required for this study. DNA concentration was measured using a spectrofluorometer, and the results were consistent, showing only minor deviations across the replicates. The PCR plate preparation involves two different master mix volumes: 19 μL for standards and 16 μL for samples (Figure 3). All standards for each gene are measured in triplicate to ensure reproducibility. Before running the PCR, the plate is centrifuged at 13,000 rpm for 6 min to ensure all components are at the bottom of the wells.

To maintain the integrity of the primers, it is crucial not to store the prepared plate for more than 30 min before running the PCR. Additionally, all samples should be gently vortexed immediately before the experiment to ensure homogeneity. For accurate results, several controls should be included in the experimental setup. A no-template control (NTC) is essential to detect any contamination, reagents degradation, or primer-dimer formation. Positive controls, both endogenous (e.g., housekeeping genes) and exogenous (known quantities of target sequence), are crucial for final concentration analysis, normalization, and assessing PCR efficiency. Endogenous controls were selected from samples that had been used in previous runs as part of the experiment.

### 2.5. Data Evaluation

All the final concentration calculations were carried out automatically in the CFX Manager software, version 3.0 (Bio-Rad Laboratories, Hercules, CA, USA). The relative quantification calibration curve result for the gene of *IFNB1* and telomeres is normalized towards endogenous control samples. Subsequently, these control values are compared and regulated across experimental plates in order to standardize the raw sample data from a plate-to-plate experiment. The baseline threshold was manually set up based on data of endogenous DNA control samples and regulated each time when the new stock of primers or standard oligomers were introduced to the experiment. Calculation of the absolute length of telomeres was carried out using the formula

$$aTL=\frac{T}{S}\;\;,$$

where

*aTL* is the absolute length of the body of telomeres; *T* is the length of the body telomeres in the kilobase; and *S* is the amount of a representative gene.

*T* and C values were determined for each sample in three repetitions using qPCR standard curve calculations for telomeres and *IFNB1*. The data analysis was performed by utilizing the CFX Manager software, version 3.0 (Bio-Rad Laboratories, Hercules, CA, USA). The calculated value was divided into 92 in order to obtain telomere length per single chromosome in a diploid set [10].

## 3. Procedure

### 3.1. Primer Dilution

1. Find the initial concentration of primers in millimoles. If the concentration of primers in the stock solution is 100 μM, it must be diluted to 10 μM for a working solution.
2. Prepare two Eppendorf tubes for the future diluted primers (forward and reverse)—stick a sticker with the inscription according to the model: “Primer name, concentration, date of dilution”.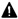 **CRITICAL STEP** all the primers should be kept on ice during the whole experiment due to risk of degradation.
4. Add 90 µL of MiQ water and 10 µL of primer stock solution to the prepared Eppendorf tube in order to obtain a 10 μM solution from 100 μM stock. Vortex gently the primers’ stock solution for 2–3 s.
5. If your concentrations differ from the example, you can calculate the volumes using the following formula:

$$V_{1}=V_{2}\frac{C_{2}}{\;C_{1}},$$

where

$V_{1}$—stock volume; $V_{2}$—final volume; $C_{2}$—final concentration; and $C_{1}$—stock concentration.

6. Store primers according to the manufacturer’s recommendations.

### 3.2. Standard Curve Preparation

1. Determine the initial concentration of oligomer standards in millimoles.
2. Label 20 Eppendorf tubes:
  - 10 tubes for Telomere: Label as “TEL 1” to “TEL 10”
  - 10 tubes for *IFNB1*: Label as “*IFNB1* 1” to “*IFNB1* 10”
3. Prepare the first standard for each oligomer. If stock concentration is 100 μM, dilute to 10 μM.

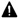
 **CRITICAL STEP** *IFNB1* oligomer stock and dilutions should be kept on ice during the whole experiment due to risk of degradation.

4. Add 90 µL of MiQ water to tube 1 of each oligomer.
5. Vortex the stock oligomer gently for 2–3 s.
6. Add 10 µL of stock oligomer to tube 1 and pipette gently 2–3 times.
7. Prepare dilution series: Add 90 µL of MiQ water to tubes 2–10 for each oligomer.
8. Create 10-fold dilution series. Start with tube 1.
9. Vortex tube 1 gently, pipette 2–3 times, and transfer 10 µL from tube 1 to tube 2.
10. Repeat this process for tubes 2–10, always transferring 10 µL from the previous tube.
11. Check concentration of oligomer in each tube utilizing the Nanodrop 1000 spectrophotometer.
12. Calibrate the NanoDrop spectrophotometer with nuclease-free water.
13. Measure 1–2 μL of each standard dilution using the NanoDrop 1000 spectrophotometer on ssDNA-33 mode.
14. Record the concentration in ng and purity (A260/280 ratio) for each dilution (Table 3).
15.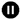 **PAUSE STEP** Store primers according to the manufacturer’s recommendations.

### 3.3. Calibration Curve Concentration Calculations

1. For telomere standard, calculate the weight of one molecule:
  - Weight of one molecule = $\frac{Molecular\;Weight}{{Avogadro}^{\prime }s\;number}$;
  - Molecular Weight (telomere) = 25,030 $\frac{g}{mol}$;
  - Weight of one molecule = $\frac{25,030}{6.022\;\times \;10^{-23}}=\;4.156\times 10^{-20}g$

2. Calculate molecules in the working stock:
  - Molecules in working stock = $\frac{Concentration\;in\;g}{Weight\;of\;one\;molecule}$;
  - Concentration of telomere in grams (standard 3 dilution by 1000× fold) = $3.11\times 10^{-9}\frac{g}{\mu L}$;
  - Molecules in working stock = $\;\frac{3.11\;\times \;10^{-9}}{4.156\times 10^{-20}}=\;7.482\times 10^{-10}\frac{molecules}{µL}$

3. Calculate concentration of telomere oligomer in kb:
  - Concentration of telomere oligomer in kb = Molecules in working stock ∗ 84 bp oligomer length;
  - Concentration of telomere oligomer in kb = $7.482\times 10^{-10}\times 84=\frac{6.285\times 10^{-12}kb}{µL}$
4. For the final concentration of the single copy gene *IFNB1*, repeat steps 1 and 2 using information from Table 2.
5. Adjust your calculations for other dilutions by multiplying or dividing by 10 accordingly.

### 3.4. Plate Layout Design

**Figure 4:**
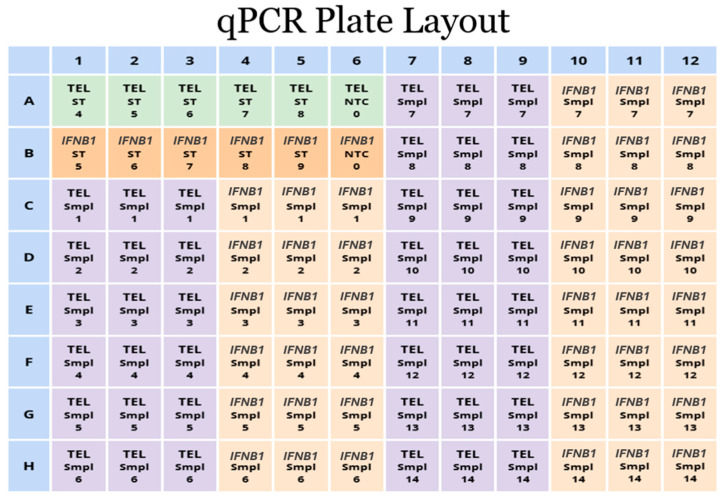
Experimental plate example layout, where ST—standard oligomer, Smpl—tested sample. Numbers in “Smpl” wells denote sample IDs run in triplicates. Green and dark orange wells indicate Telomere and *IFNB1* standards, with numbers representing the dilution step. Purple and light beige wells distinguish samples amplified with Telomere and *IFNB1* specific primers, respectively.

1. Create a new plate layout in the CFX Manager software, version 3.0 (Bio-Rad Laboratories, Hercules, CA, USA). The experimental plate example layout is demonstrated in Figure 4.

2. Assign wells for standard oligomers for telomere from tube 4 to 8 and for *IFNB1* from tube 5 to 9.
3.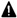 CRITICAL STEP: adjust your calibration choice based on your individual concentration measurements for standard oligomers and run each standard in triplicate if necessary.
4. Add DNA samples in triplicate.
5. Include no-template controls (NTC) and endogenous control samples using an inhouse DNA template or previously run samples with known results.
6. Each endogenous control should be tested in triplicate, with at least one included in your experiment. Incorporating more endogenous controls in a single run can improve the precision of your results; at the same time, it will also reduce the number of wells available for your screening samples.
7. Put calculated concentrations of standard oligomers for telomere in kb and for *IFNB1* standard in the copy number.

### 3.5. Master Mix Calculation

1. Open the Master Mix Calculator in the CFX Manager software, version 3.0 (Bio-Rad Laboratories, Hercules, CA, USA).
2. Enter the following parameters for two MasterMixes for standard oligomer and DNA sample templates for each of the genes (Telomere and *IFNB1*):
  - Total reaction volume: 20 μL;
  - Template volume (for standard oligomers): 1 μL;
  - Template volume (for samples) 4 μL (20 $\frac{ng}{\mu L}$);
  - Final primer concentration in MasterMix: 100 nM;
  - Starting Concentration forward and reverse primer 10 μM;
  - Mix volume without template for sample: 16 μL;
  - Mix volume without template for standard: 19 μL;
  - 5× qPCRmix-HS SYBR (Evrogen, Moscow, Russia, PK147S) with HotStart Polymerase.
3. Calculate separate master mixes for Telomere and *IFNB1* standards-4.
4. MasterMixes total—2 for Telomere and 2 for *IFNB1* standard.
5. Primers’ concentration calculation in summary:
  - Primer stock: 100 μM;
  - Working solution: 10 μM (prepared by 1:10 dilution of stock);
  - Final concentration in PCR: 100 nM (using 0.2 μL of 10 μM primers in 20 μL total reaction volume).

### 3.6. qPCR Conditions and Set Up

1. Prepare and label 4 Eppendorf 2 mL tubes as follows: MM TEL Standards, MM TEL Samples, MM *IFNB1* Standards, MM *IFNB1* Samples.
2. Add reagents to each Master Mix tube in the following order: Fresh MiQ Water, 5× qPCRmix-HS SYBR, Forward primers, Reverse primers.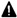 CRITICAL STEP Change pipette tips after adding each component.
4. Place a 96-well plate on an ice tray or an ice box to use as a holder for 0.2 mL thin-walled PCR tubes.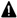 CRITICAL STEP Keeping PCR tubes cold helps prevent nuclease activity, primer dimer formation, reagent degradation, and nonspecific priming.
6. Pipette PCR reagents into each 0.2 mL PCR well in the following order: Master Mix (MM Standards 19 μL and MM Samples 16 μL), then standard oligomers (1 μL) and samples (4 μL).
7. Prepare your plate and centrifuge at 13,000 rpm for 6 min.
8. Set up preincubation for 3 min at 95 °C; then 40 cycles including denaturation at 95 °C for 15 s, at 60 °C for 20 s, at 72 °C for 10 s (signal acquisition), and 60 °C for 30 s (melt curve).

### 3.7. Results Calculations and Analysis

1. Check experimental regression factor: R^2^ should be bigger than 95% or at least 90%, and efficacy should be ±10% from 100%.
2. Use the CFX Manager software, version 3.0 (Bio-Rad Laboratories, Hercules, CA, USA) to automatically calculate the Cq Mean concentrations of telomeres and *IFNB1* based on the calibration curve calculations.
3. Normalize the relative quantification results of telomeres (T) and *IFNB1* (S) to endogenous control samples.
4. Standardize these normalized values across experimental plates to account for plate-to-plate variability.
5. Manually set a baseline threshold for each experimental plate based on the data from endogenous DNA control samples.
6. Perform average quantification analysis of sample triplicates.
7. For each sample, determine *T* (telomere length in kilobases) and *S* (amount of reference gene *IFNB1* in copy number) values in three technical replicates using qPCR standard curve calculations.
8. Calculate *aTL* use the formula:

$$aTL=\frac{T}{S},$$

where:

*T*: Telomere length in kilobases, calculated from PCR results.

*S*: Amount of reference gene *IFNB1*, calculated from PCR results.

9 OPTIONAL STEP Adjust your calculations for the diploid chromosome set by dividing the calculated *aTL* by 92 to obtain the telomere length per single chromosome in a diploid set: Telomere Length Per Chromosome $=\;\frac{aTL}{92}.$
10. Perform all data analysis using the CFX Manager software, version 3.0 (Bio-Rad Laboratories, Hercules, CA, USA) to ensure consistency and accuracy in calculations.
11. Report your final telomere length value.

**Notes**

- This technique is highly susceptible to contamination, particularly from synthesized primers and oligonucleotides;
- The quality in manufacturing of primers and oligonucleotides is crucial;
- To prevent degradation, it is advisable to keep all primers and oligonucleotides on ice throughout the experiment;
- Primers are particularly sensitive to temperature fluctuations. To reduce the frequency of freeze-thaw cycles, creating multiple small volume dilution preparations primers is recommended, which will help avoid degradation and the formation of dimers;
- Additionally, the quality of PCR is influenced by the concentration of sample DNA; amplification results can vary significantly due to inaccurate measurements and substantial differences in concentration between samples;
- If your *IFNB1* Ct values fluctuate by more than 3–4 cycles, it may indicate unequal sample concentrations, primer dimers, or degradation.
- For samples with a calculated telomere length or *IFNB1* copy number that falls outside the range of the primary standard curve, additional dilutions of the standard curve must be performed to ensure that the sample values are within the calibration range. Measurements taken near the detection limits are recorded taking into account the amplification efficiency and potential variability to avoid misinterpretation.

## 4. Results

The results presented in Table 4 highlight the measurements of aTL across 17 adult volunteer samples and 9 samples from healthy newborns, alongside related parameters such as telomere length in kilobases, *IFNB1* copy number, and age. For adults, the aTL values ranged from 4.29 × 10^0^ kb to 8.31 × 10^1^ kb, with corresponding values for aTL per chromosome varying between 4.67 × 10^−2^ kb and 9.03 × 10^−1^ kb. These results indicate significant variability in telomere length among individuals, with no clear correlation observed between age and telomere length within this dataset. For example, sample 5 (age 27) exhibited the highest aTL (8.31 × 10^1^ kb), while sample 7 (age 27) showed one of the lowest aTL values (4.29 × 10^0^ kb).

Additionally, telomere length (total signal in kilobases) varied significantly across samples, ranging from 9.23 × 10^5^ to 4.46 × 10^7^, suggesting individual differences in genomic stability or telomere dynamics [12]. The *IFNB1* copy number also showed variation, spanning from 1.09 × 10^5^ to 1.53 × 10^6^, which may influence telomere maintenance mechanisms or cellular responses.

For newborn babies, the aTL in kb was substantially higher, ranging from 9.94 × 10^2^ to 2.83 × 10^3^, with aTL per chromosome varying between 1.08 × 10^1^ and 3.07 × 10^1^. Newborn samples exhibited some of the highest telomere lengths in the study (e.g., sample 21 had 1.29 × 10^8^ kb, the highest total signal in the dataset). Their *IFNB1* copy numbers were relatively low compared to adult samples, generally ranging from 2.91 × 10^4^ to 8.20 × 10^4^.

**Figure 5:**
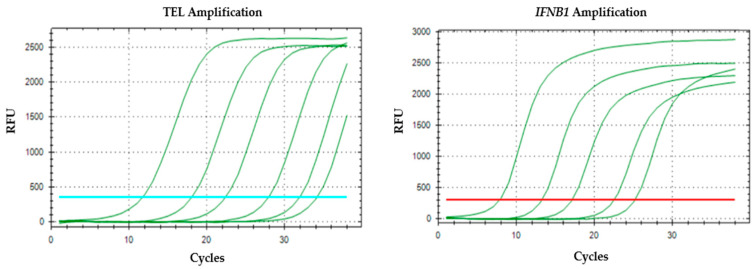
qPCR amplification plots for the standard dilution calibration curve. The graphs illustrate the amplification of telomere (TEL) (standards 4 to 8) and *IFNB1* sequences (standards 5 to 9), shown as the first five green curves from left to right. The sixth curve represents the NTC. The horizontal blue and red lines indicate the thresholds determined automatically by the software.

The qPCR amplification graphs for the standard dilution calibration curve illustrate the amplification of TEL and *IFNB1* sequences over successive cycles. Both graphs demonstrate high efficiency and specificity of the qPCR reactions, as evidenced by the clear separation between curves for different dilutions and consistent exponential amplification phases. These calibration curves are essential for quantifying aTL by comparing unknown samples against known standards (Figure 5).

**Figure 6:**
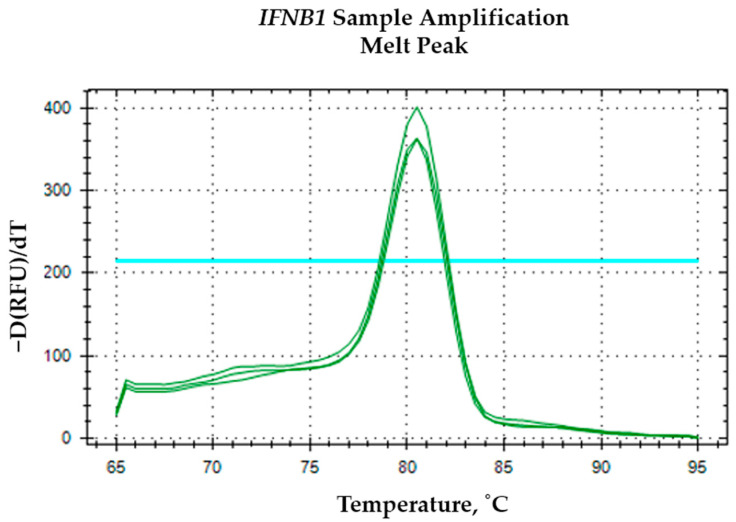
The green melting curve for *IFNB1* endogenous control sample amplification. The horizontal blue line indicates the threshold determined automatically by the software.

**Figure 7:**
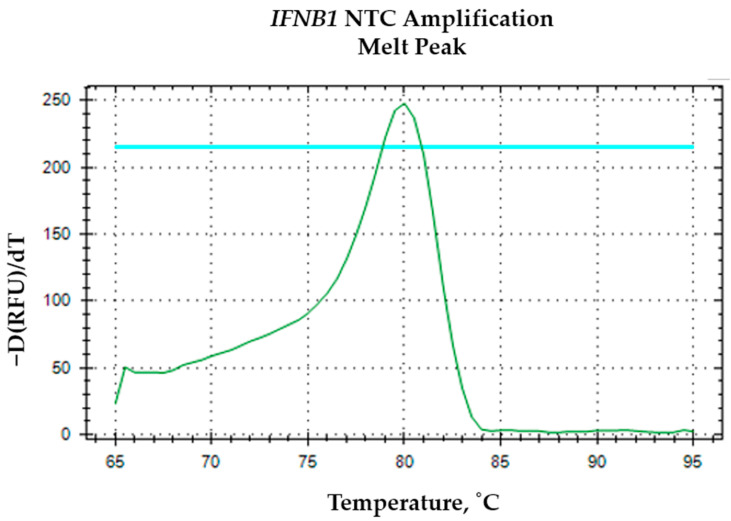
The green melting curve for *IFNB1* NTC amplification. The horizontal blue line indicates the thresholds determined automatically by the software.

The *IFNB1* oligomer curves in Figure 5 do not reach the same maximum fluorescence levels as the first and second telomere repeat oligomers; their amplification does not fully extend to the fluorescence plateau observed with the telomere repeat curves. It can be explained by several factors related to amplification efficiency and molecular biology of the targets. Telomere repeats are highly repetitive sequences, allowing for more efficient and robust amplification during qPCR or similar fluorescence-based assays. This repetitive nature leads to a higher yield of amplicons per cycle, which in turn results in a more rapid and complete increase in fluorescence signal, often reaching the maximum detectable level within fewer cycles [13]. In contrast, *IFNB1* amplification efficiency can be inherently lower due to sequence complexity, which can limit the extent of fluorescence amplification. The specificity of the IFNB1 amplification was further verified by melt curve analysis, which displayed a distinct single peak for samples (Figure 6) and no specific signal in the NTC (Figure 7).

The Table 5 presents aTL data measured in kilobases and normalized per chromosome for buccal swab and blood samples from the same individuals. The results show variability between sample types: in Sample 1, buccal cells have longer telomeres (65.7 kb) than blood (34.0 kb), while Sample 2 shows nearly identical aTL values for both tissues (~37.4 kb). For Samples 3 and 4, telomere length is notably longer in blood (61.3 kb and 10.6 kb, respectively) compared to buccal cells (4.63 kb and 3.39 kb). The aTL per chromosome values align with these patterns, reflecting tissue-specific differences. Although ages range from 23 to 38 years, no clear age-related trend in telomere length can be observed from this limited data set.

## 5. Discussion

This study provides a comprehensive analysis of aTL across 26 DNA samples extracted from whole blood cells, alongside related parameters such as telomere length in kilobases, *IFNB1* copy numbers, and age. The results reveal significant variability in telomere length among individuals, with aTL values ranging from 6.43 × 10^−2^ kb to 3.07 × 10^1^ kb to corresponding aTL-per-chromosome values. Despite the expectation that age would correlate with telomere shortening, no clear relationship between chronological age and telomere length was observed in this dataset.

This study builds upon the foundational work of O’Callaghan [10], who developed a monoplex one-plate two-reaction tube protocol for simultaneous amplification of telomeres and single-copy genes under identical cycling conditions. While their protocol employed the *36B4* reference gene, which is not a true single-copy gene [14], our approach adopted *IFNB1* as a genuine single-copy reference gene to improve reliability.

Additionally, we implemented further modifications to improve standard curve generation. Unlike the Siegel SR et al. method that utilized double-stranded oligomers [11]—which are often challenging to manufacture consistently—we opted for single-strand antisense oligonucleotides as standards due to difficulties in synthesizing double-stranded standards and achieving stable results with in-house annealing protocols. This approach allowed us to bypass these limitations while maintaining reliable quantification.

Buccal swabs collect epithelial cells from the inner cheek, which are easy to obtain but may produce variable DNA quality and quantity due to factors like collection technique, cellular material amount, and saliva contamination [15]. These buccal cells are mostly terminally differentiated with slower turnover, which can lead to DNA degradation or reduced yield, affecting PCR accuracy and potentially causing underestimation of telomere length [16]. In contrast, blood samples primarily contain leukocytes that renew continuously, maintaining more consistent and often longer telomere lengths because of ongoing cell replication. The slower renewal and aging of buccal cells compared to dynamically replenished blood cells contribute to the differences observed in telomere length measurements between the two sample types.

The primers used in this study were specifically designed and validated for Homo sapiens sequences and are not intended for, nor optimized to amplify, DNA from other species.

It is important to note that in this protocol, Ct values for the single copy gene (*IFNB1*) often appear earlier than Ct values for telomeres in adult human samples. This contrasts with the relative qPCR method described by Cawthon [17], where *36B4* is typically amplified later than telomeres.

This difference in amplification time is not related to non-specific amplification of *IFNB1*, which is confirmed by a single peak in the melting curves and stable kinetic patterns observed on pure synthetic oligonucleotide standards that do not contain genomic DNA. Instead, this shift is the result of the specific thermodynamic conditions of our analysis. Unlike previous protocols using high primer concentrations (up to 900 nM) and lower annealing temperatures (54 °C for 2 min) to accelerate the hybridization of telomeric primers with mismatches, our method uses harsh conditions (100 nM primers, annealing at 60 °C for 20 s). Under these conditions, fully complementary *IFNB1* primers initiate amplification with higher kinetic efficiency than telomeric primers, which naturally leads to an earlier crossing of the threshold for *IFNB1*.

In addition, the variability of ∆Ct (Ct Tel − Ct (*IFNB1*)) in our samples reflects natural biological differences: in newborns with long telomeres, telomere amplification may outpace *IFNB1* (negative Ct), whereas in adults the opposite pattern is usually observed.

Thus, the earlier yield of *IFNB1* reflects the high specificity and effectiveness of the selected primers under harsh reaction conditions. The use of the standard curve method fully compensates for these kinetic features, ensuring measurement accuracy regardless of the order in which the fluorescence signals appear.

In conclusion, this study highlights the heterogeneity of telomere length among individuals and presents an improved qPCR-based protocol for measuring absolute telomere length using *IFNB1* as a reference gene and single-strand standards. These findings address key methodological limitations of prior approaches while providing valuable insights into telomere biology. Future studies should focus on refining these methods further and exploring additional genetic and environmental factors influencing telomere maintenance mechanisms to enhance our understanding of their role in health and disease.

## Figures and Tables

**Table 1:** The sequence of primers for Telomere and *IFNB1*.

Primer	Sequence	Amplicon Size
FTM	CGG TTT GTT TGG GTT TGG GTT TGG GTT TGG GTT TGG GTT	76 bp
RTM	GGC TTG CCT TAC CCT TAC CCT TAC CCT TAC CCT TAC CCT	
*IFNB1* Fexon	CAACAGGTAGTAGGCGACAC	83 bp
*IFNB1* Rexon	GAGAAGCACAACAGGAGAGC	

bp—base pair

**Table 2:** The sequences, size, and molecular weight of oligomer standards.

Oligomer Standard	Sequence	Size	Molecular Weight
Telomere	(TTAGGG)14	84 bp	25,030
*IFNB1* exon antisense	GAGAAGCACAACAGGAGAGCAATTTGGAGGAGACACTTGTTGGTCATGTTGACAACACGAACAGTGTCGCCTACTACCTGTTG	83 bp	25,811

**Table 3:** Analytical sensitivity of the real-time PCR systems for standard curve oligomer dilution.

Dilution of the Standard Oligomer	Cq Values	Starting Quantity	Oligomer
10^−4^	12.45	6.140 × 10^8^ kb	
10^−5^	16.51	6.140 × 10^7^ kb	
10^−6^	19.61	6.140 × 10^6^ kb	Telomere
10^−7^	24.30	6.140 × 10^5^ kb	
10^−8^	28.01	6.140 × 10^4^ kb	
10^−5^	4.12	9.890 × 10^8^ cn	
10^−6^	8.08	9.890 × 10^7^ cn	
10^−7^	12.02	9.890 × 10^6^ cn	*IFNB1*
10^−8^	16.51	9.890 × 10^5^ cn	
10^−9^	20.37	9.890 × 10^4^ cn	

kb—kilobase; cn—copy number.

**Table 4:** Final data aTL analysis based on PCR experimental results of telomere and *IFNB1* in blood samples.

Sample Number	Telomere in kb	*IFNB1* in Copy Number	aTL in kb	aTL Per One Chromosome in kb	Age
1	3.86 × 10^6^	1.09 × 10^5^	3.54 × 10^1^	3.85 × 10^−1^	29
2	2.28 × 10^6^	1.47 × 10^5^	1.55 × 10^1^	1.69 × 10^−1^	34
3	1.47 × 10^7^	1.53 × 10^6^	9.61 × 10^0^	1.04 × 10^−1^	23
4	1.31 × 10^6^	3.03 × 10^5^	4.32 × 10^0^	4.70 × 10^−2^	24
5	2.06 × 10^7^	2.48 × 10^5^	8.31 × 10^1^	9.03 × 10^−1^	27
6	9.23 × 10^5^	2.09 × 10^5^	4.42 × 10^0^	4.80 × 10^−2^	28
7	5.84 × 10^6^	1.36 × 10^6^	4.29 × 10^0^	4.67 × 10^−2^	27
8	1.07 × 10^6^	1.81 × 10^5^	5.91 × 10^0^	6.43 × 10^−2^	28
9	6.25 × 10^6^	3.94 × 10^5^	1.59 × 10^1^	1.72 × 10^−1^	24
10	9.51 × 10^5^	2.12 × 10^5^	4.49 × 10^0^	4.88 × 10^−2^	23
11	3.62 × 10^6^	2.51 × 10^5^	1.44 × 10^1^	1.57 × 10^−1^	23
12	2.09 × 10^6^	3.57 × 10^5^	5.85 × 10^0^	6.36 × 10^−2^	23
13	5.89 × 10^6^	1.58 × 10^5^	3.73 × 10^1^	4.05 × 10^−1^	29
14	2.23 × 10^6^	1.30 × 10^5^	1.72 × 10^1^	1.86 × 10^−1^	28
15	4.46 × 10^7^	6.19 × 10^5^	7.21 × 10^1^	7.83 × 10^−1^	22
16	1.77 × 10^7^	5.59 × 10^5^	3.17 × 10^1^	3.44 × 10^−1^	42
17	3.11 × 10^7^	5.53 × 10^5^	5.62 × 10^1^	6.11 × 10^−1^	24
18	3.91 × 10^7^	3.86 × 10^4^	1.01 × 10^3^	1.10 × 10^1^	0
19	8.12 × 10^7^	2.96 × 10^4^	2.74 × 10^3^	2.98 × 10^1^	0
20	9.74 × 10^7^	7.29 × 10^4^	1.34 × 10^3^	1.45 × 10^1^	0
21	1.29 × 10^8^	7.54 × 10^4^	1.71 × 10^3^	1.86 × 10^1^	0
22	8.03 × 10^7^	8.08 × 10^4^	9.94 × 10^2^	1.08 × 10^1^	0
23	8.23 × 10^7^	2.91 × 10^4^	2.83 × 10^3^	3.07 × 10^1^	0
24	1.03 × 10^8^	8.20 × 10^4^	1.26 × 10^3^	1.37 × 10^1^	0
25	8.16 × 10^7^	3.24 × 10^4^	2.52 × 10^3^	2.74 × 10^1^	0
26	7.56 × 10^7^	2.91 × 10^4^	2.60 × 10^3^	2.82 × 10^1^	0

**Table 5:** Data aTL analysis showing buccal swab and blood samples based on PCR experimental results of Telomere and *IFNB1*. Buccal- buccal swab, blood-peripheral blood sample. A sample with the same number contains the biomaterial from the same person.

Sample Number	Telomere in kb	*IFNB1* in Copy Number	aTL in kb	aTL per One Chromosome in kb	Age
1 buccal	6.24 × 10^5^	9.50 × 10^3^	6.57 × 10^1^	7.14 × 10^−1^	29
1 blood	6.12 × 10^6^	1.80 × 10^5^	3.40 × 10^1^	3.70 × 10^−1^	
2 buccal	4.53 × 10^6^	1.21 × 10^5^	3.74 × 10^1^	4.07 × 10^−1^	23
2 blood	4.67 × 10^6^	1.25 × 10^5^	3.74 × 10^1^	4.06 × 10^−1^	
3 buccal	1.82 × 10^6^	3.93 × 10^5^	4.63 × 10^0^	5.03 × 10^−2^	27
3 blood	1.36 × 10^7^	2.22 × 10^5^	6.13 × 10^1^	6.66 × 10^−1^	
4 buccal	5.52 × 10^5^	1.63 × 10^5^	3.39 × 10^0^	3.68 × 10^−2^	38
4 blood	4.00 × 10^6^	3.78 × 10^5^	1.06 × 10^1^	1.15 × 10^−1^	

## Data Availability

The data that support the findings of this study are available on request from the corresponding author. The data are not publicly available due to privacy or ethical restrictions.

## Supplementary Materials

The following supporting information can be downloaded at: https://www.mdpi.com/article/10.3390/mps9010022/s1, S1: Short review of other Telomere Length Estimation PCR Methods. S2: Duplex IFNB1 and Telomere oligomere analysis results. Figure S1: The qPCR amplification graphs for the standard dilution calibration curve *IFNB1* duplex from standard 5 to standard 9; Figure S2: The Melting Curve amplification graphs for the standard dilution calibration curve *IFNB1* duplex amplification from standard 5 to standard 9; Figure S3: The qPCR amplification graphs for the standard dilution calibration curve telomere (TEL) duplex from standard 4 to standard 8 from left to right starting with standard 4; Figure S4: The Melting Curve amplification graphs for the standard dilution calibration curve telomere (TEL) duplex amplification from standard 4 to standard 8.

## Abbreviations

qPCR	quantitative Real-Time Polymerase Chain Reaction
rTL	relative telomere length
aTL	Absolute telomere length
*IFNB1*	Interferon beta 1
bp	Base pair
kb	kilobase
cn	Copy number
