# Supplementary material for: Improved Step-by-Step qPCR Method for Absolute Telomere Length Measurement
Source: Methods Protoc. 2026 Feb 5;9(1):22. doi: 10.3390/mps9010022 (PMC12921970; doi:10.3390/mps9010022)
Supplement: Supplementary file 1 [file mps-09-00022-s001.zip › mps-4010510-supplementary.pdf]

Supplementary

1. Short review of other Telomere Length Estimation PCR Methods

Cawthon 2002

Although this method is old and has a few disadvantages, it is still widely used across labs to measure relative telomere length [1]. The main obstacle of the Cawthon 2002 method is the reference gene - *36B4*. At the time of protocol development, it was believed to be a single copy gene. However, recent evidence has shown that *RPLP0* (*36B4*) represents a standard ribosomal gene accompanied by numerous processed pseudogenes within the human genome. Even though it is possible to adapt the final T/S calculation to the number of *36B4* levels of amplification – it still gives invalid results and can create some errors in estimation. The next crucial disadvantages of this method are experimental variability and time consumption. The Cawthon 2002 method uses two separate cycling conditions for telomere and scg amplification, meaning you are using two various plates, which are either taken at different times on the same PCR equipment or done simultaneously on different amplification systems. It creates a time gap and confounding variables, which makes the experimental results invalid and the whole method time consuming. However, there are a few reasons why modern labs still prefer to use the Cawthon 2002 method. Firstly, it is easier to adapt to the different lab conditions and equipment compared to other methods (see supplemental materials), including the PCR machine model, primer design and efficacy, and time-consumption for validation. Moreover, this method is old, thus a lot of troubleshooting suggestions were implemented through time.

Cawthon 2009

The second most popular method in telomere length estimation is Cawthon 2009 [2]. This method allows for the simultaneous quantification of two distinct amplicons within a single reaction tube, despite their significant variations in copy numbers and melting temperatures. Specifically, the first target (designated as T), which is identified as the more abundant species, produces detectable signals at earlier cycles, as indicated by its lower threshold cycle (CT) values. Conversely, the signal for the less abundant target (referred to as S) remains at the baseline level during these initial cycles. The reference gene (scg) was changed to hbb and albumin, which satisfy the condition of a single copy gene. The one-reaction tube feature is enhancing the validity of the experimental outcomes by lowering its variability. Furthermore, the performance of this protocol requires a lesser amount of plastic plates and tubes and consumes a lower amount of time for the whole experiment. At the same time, some labs may have trouble with two-signal detection as not all cycling equipment and cycling data collection software allow it or can make it correctly. Furthermore, during cycling reactions, the MasterMix reagents can be expended unevenly between two amplifications—telomere and single copy gene—due to the one-reaction tube protocol. It also brings the issues of PCR efficacy. Additionally, this method shows some troubles with hbb dimers' formation, and the overall method of lab adaptation is time consuming.

O'Callaghan 2011

This method represents a monoplex one-plate two-reaction tube protocol, meaning that amplification of telomere and scg is occurring simultaneously under the same cycling conditions [3]. Although the procedure is unreliable due to the use of *36B4* as a reference gene, this protocol has inspired the line of this method of optimization papers, where *36B4* is replaced with *IFNB1* or actin. The debatable point of this method is the use of plasmids to satisfy the DNA amount added within standard dilutions for telomeres and reference genes. Additionally, this method is modification-friendly, which provides a good base for trouble-shooting moderations. Siegel et al. in 2023 proposed a significant modification of this protocol, which resulted in a good detailed review of the method and a new view of its adaptation.

Siegel 2023

A significant alteration of O'Callaghan 2011 in this method [4] was the use of a truly single copy reference gene *IFNB1*, compared to previous protocols of colleagues. The use of double-stranded oligomere

for standard curve formation is an innovative step and was created to simulate more realistic conditions for DNA samples for DNA amplification reactions. Although this step of the method is less adaptable across various labs due to country-specific oligomer availability from commercial manufacturers for double-stranded synthesized DTO standards.

The authors used a sense and antisense sequence of standards in one test tube for both telomeres and interferon, as well as plasmids and no plasmids. As in the O'Callaghan protocol, simultaneous amplification remains in one mode, but so far, the task of setting a multiplex reaction remains unresolved. In our protocol modification, single-strand antisense oligonucleotides are used as standards due to the absence of double-strand manufacture and the lack of stable results after numerous trials of performing an inhouse annealing protocol for connecting two separately synthesized antisense and sense telomere standards.

2. Duplex *IFNB1* and Telomere oligomere analysis results.

Duplex structures and primers proposed by Siegel S.R were tested in our laboratory conditions [4]. The preliminary mixing of sense and antisense standards with heating caused an incorrect construction of the standard curve, which may be due to the formation of supramolecules. Therefore, the standards must be added separately, which does not simplify the preparation for PCR and also introduces some error during pipetting. The duplex interferon standards gave a very strong relative fluorescence signal, while the telomere gave a signal below the single variant.

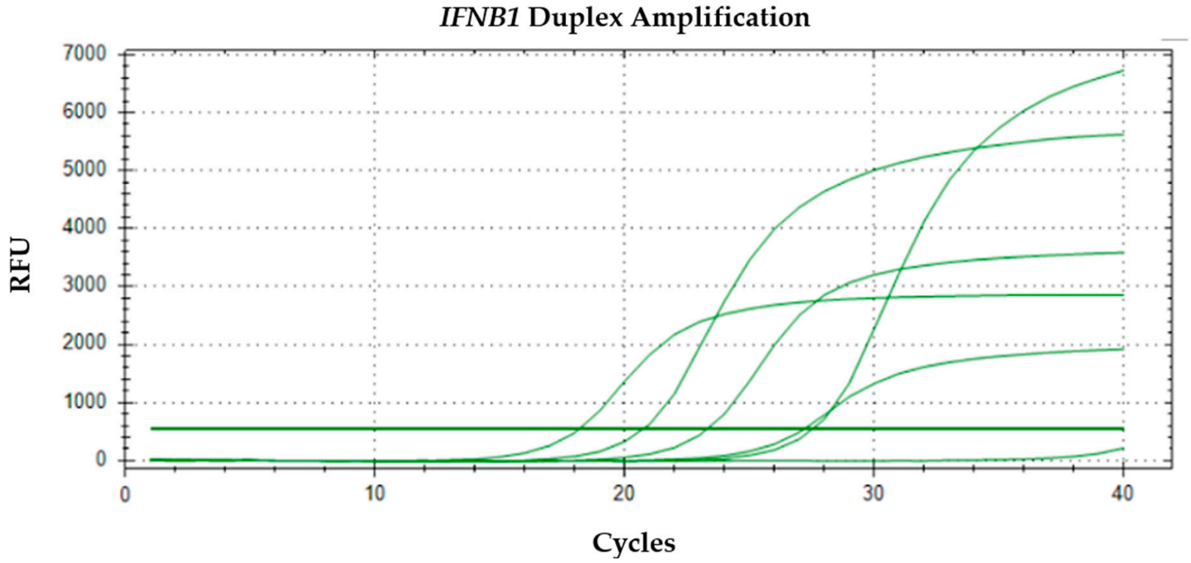

Figure S1. The qPCR amplification graphs for the standard dilution calibration curve *IFNB1* duplex from standard 5 to standard 9. NTC from left to right, starting with standard 5.

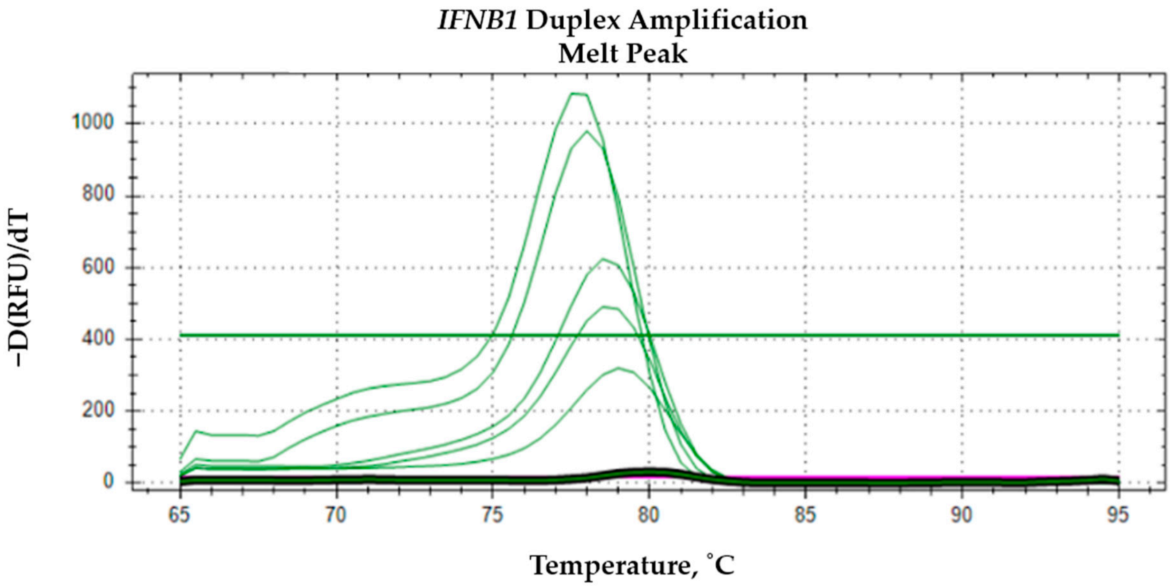

Figure S2. The Melting Curve amplification graphs for the standard dilution calibration curve IFNB1 duplex amplification from standard 5 to standard 9. NTC is represented by thick dark green line.

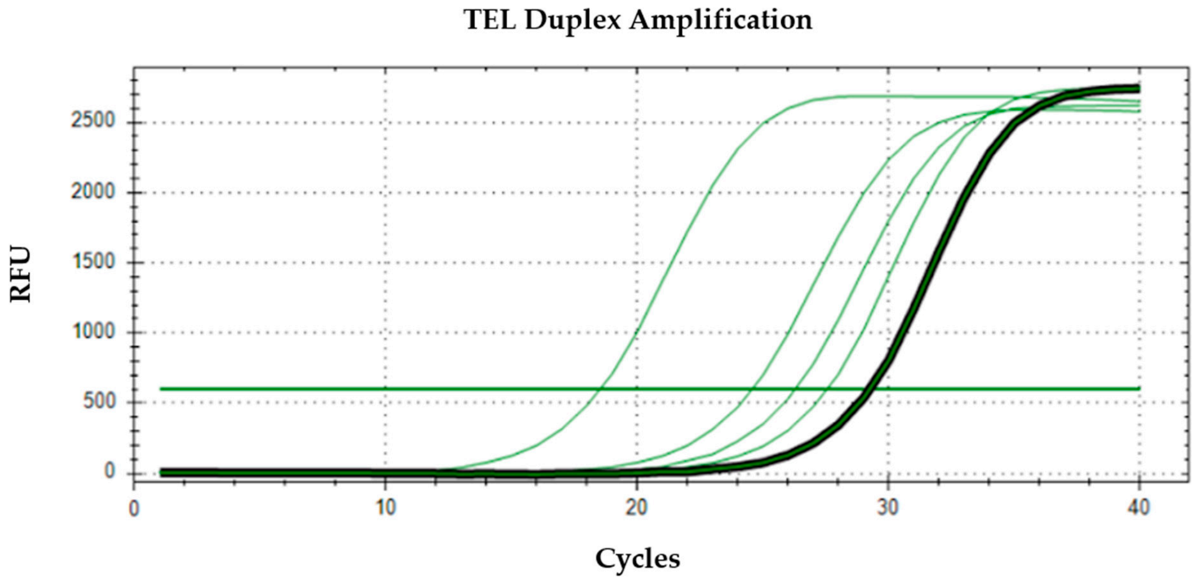

Figure S3. The qPCR amplification graphs for the standard dilution calibration curve telomere (TEL) duplex from standard 4 to standard 8 from left to right starting with standard 4. The thick green line represents standard 8.

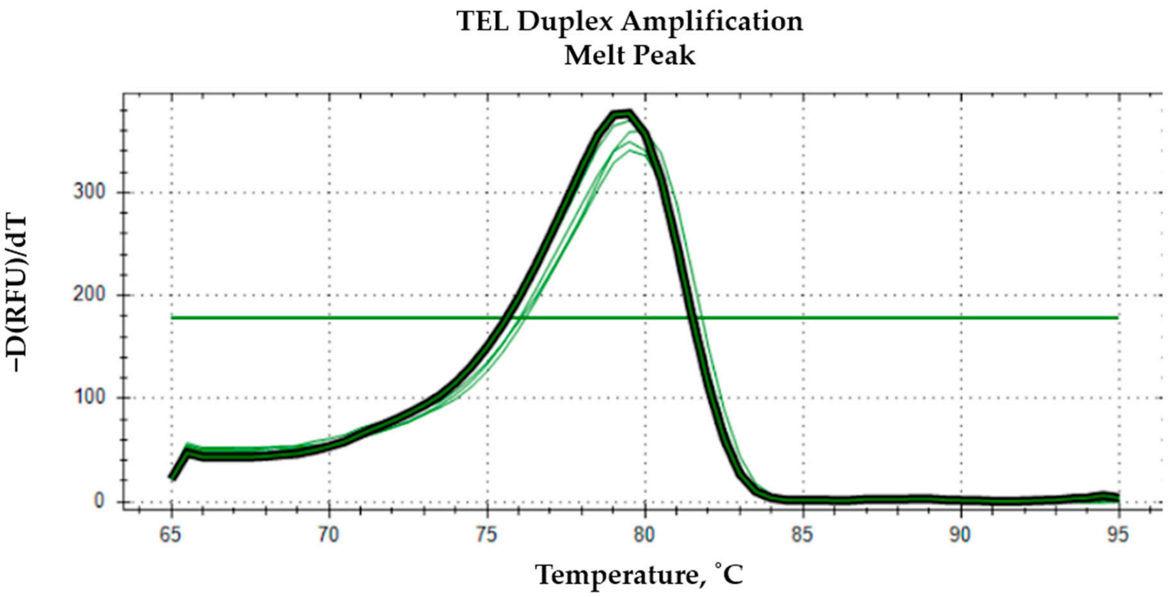

Figure S4. The Melting Curve amplification graphs for the standard dilution calibration curve telomere (TEL) duplex amplification from standard 4 to standard 8. The thick green line represents standard 8.

Our approach to generating duplex molecules from single-stranded sense and antisense oligomers utilized three distinct methods: (1) directly adding the individual oligomers separately into the PCR plate, allowing duplex formation to occur in situ; (2) pre-mixing equal volumes and concentrations of sense and antisense oligomer stock solutions (for example, combining 50 µl of each at 100 µM) prior to use; and (3) pre-mixing the oligomers followed by a brief preheating step at 95°C for 3–5 minutes using a thermoshaker to promote efficient annealing before further processing. The first method yielded inconsistent results and an unreliable calibration curve, whereas the latter two approaches produced consistent unsuccessful outcomes, as illustrated in Figures 1–3.

References

1. Cawthon, R.M. Telomere measurement by quantitative PCR. *Nucleic Acids Res.* **2002**, *30*, e47. https://doi.org/10.1093/nar/30.10.e47. PMID: 12000852; PMCID: PMC115301.
2. Cawthon, R.M. Telomere length measurement by a novel monochrome multiplex quantitative PCR method. *Nucleic Acids Res.* **2009**, *37*, e21. https://doi.org/10.1093/nar/gkn1027. PMID: 19129229; PMCID: PMC2647324.
3. O'Callaghan, N.J.; Fenech, M. A quantitative PCR method for measuring absolute telomere length. *Biol. Proced. Online* **2011**, *13*, 3. https://doi.org/10.1186/1480-9222-13-3. PMID: 21369534; PMCID: PMC3047434.
4. Siegel, S.R.; Ulrich, M.; Logue, S.F. Comparison qPCR study for selecting a valid single copy gene for measuring absolute telomere length. *Gene* **2023**, *860*, 147192. https://doi.org/10.1016/j.gene.2023.147192. PMID: 36641077.
